# Patterns of rest-activity rhythms from adolescence to young adulthood: a scoping review

**DOI:** 10.3389/frsle.2026.1779808

**Published:** 2026-04-08

**Authors:** Camila Koike, Bridget A. Nestor, Erin Sands, Danielle A. Wallace, Joe Kossowsky

**Affiliations:** 1 Department of Anesthesiology, Critical Care, and Pain Medicine, Boston Children's Hospital, Boston, MA, United States; 2 Department of Anesthesia, Harvard Medical School, Boston, MA, United States; 3 Department of Psychology, Endicott College, Beverly, MA, United States; 4 Gangarosa Department of Environmental Health, Rollins School of Public Health, Emory University, Atlanta, GA, United States; 5 Division of Sleep Medicine, Harvard Medical School, Boston, MA, United States

**Keywords:** actigraphy, adolescents, predictors, rest-activity rhythms, wearables, young adults

## Abstract

**Introduction:**

Rest-activity rhythms (RAR) measure daily physical activity patterns. RAR metrics correlate with biopsychosocial trajectories in adults and may represent objective biomarkers of health and risk. However, the significance of RAR to demographical, physical, and psychological factors in early adolescence and young adulthood has not been collectively evaluated, leaving gaps in understanding RAR's relevance during this developmental window.

**Methods:**

A systematic search identified peer-reviewed studies on adolescents and young adults (ages 10–19) that reported data on RAR variables and factors of interest published through April 2024. Study and participant characteristics, device characteristics, days of wearable data collected, RAR variables, and associated demographic, social, physical, and psychological factors were extracted.

**Results:**

Of 1,648 screened titles and abstracts, 19 studies with 16,717 participants met eligibility. Included studies varied widely in devices used, epoch lengths, recording duration, and RAR data compliance criteria. Seven studies reported RAR outcomes relevant to demographic or social factors, nine studies reported RAR outcomes relevant to physical health, and ten studies reported RAR results relevant to psychological factors. Across domains, findings were highly heterogeneous and differed from the more consistent and established associations observed in the adult literature. Generally, age-related decreases in stability and fragmentation were observed, as well as associations between increased fragmentation and cardiometabolic risk and between reduced peak daytime activity and poor mental health. Consistently, younger age groups exhibited higher interdaily stability and intradaily variability, and elevated cardiometabolic risk was associated with a delayed acrophase. Methodological inconsistencies and low-quality studies limit the generalizability of findings across studies.

**Conclusion:**

Standardization in RAR measurement, establishment of age- and sex-specific normative values for RAR, and longitudinal studies with more diverse samples are necessary to advance this field and better clarify clinically relevant RAR associations.

## 1 Introduction

Rest-activity rhythms (RAR) are behavioral activity patterns that can be measured via wearable devices which collect data continuously and non-invasively using accelerometer sensors with minimal participant input. RAR metrics can be categorized into parametric and non-parametric variables, each describing distinct aspects of rest-activity behaviors (Gao et al., 2023; Wallace et al., 2023) (see Table 1). Together, they quantify the stability and variation in a person's day-to-day activity levels and provide insight into the timing of key physiological functions (including sleep and physical activity) and circadian rhythms. RAR metrics are also associated with a range of demographic, physical health, and psychological factors, potentially making them objective biomarkers of health and risk.

**Table 1:** Description of rest-activity rhythms (RAR) variables.

RAR variable	Parametric/non-parametric	Definition
Interdaily stability (IS)	Non-parametric	Quantifies the regularity of activity patterns across multiple days, indicating the consistency of these patterns from one 24-h period to the next. Higher IS values suggest a more regular sleep-wake pattern.
Intradaily variability (IV)	Non-parametric	Qualifies the fragmentation of circadian rhythms measured within 24-h period. Higher IV values suggest a more fragmented rest-activity pattern and lower variables suggest more consolidated periods of rest and activity.
Lowest 5 h (L5)	Non-parametric	Average of amplitude and timing of the first 5 consecutive hours of the lowest activity within a 24-h period. Lower L5 suggests more restful and consolidated RAR.
Highest 10 h (M10)	Non-parametric	Average of amplitude and timing of the first 10 consecutive hours of the highest activity within a 24-h period. Higher M10 suggests more robust and consolidated periods of activity, while lower values may suggest reduced activity levels or fragmented activity patterns.
Relative amplitude (RA)	Non-parametric	Calculated by the difference between M10 and L5, with higher amplitude indicating a stronger rhythmicity.
Circadian amplitude/amplitude (CA)	Parametric	Calculated by fitting a 24-h cosine curve to activity data and extracting the amplitude parameter, which reflects the magnitude of daily oscillations. Higher CA indicates a more pronounced amplitude (i.e., clear distinctions between active and rest periods), while a lower amplitude suggests a rhythm with lower variability.
Midline estimating statistic of rhythm (MESOR)	Parametric	Average activity level over a 24-h period. A higher score indicates increased activity levels throughout the day.
Acrophase	Parametric	Calculated by fitting a 24-h cosine curve to activity data and extracting the phase-shift parameter, which indicates the specific time within a 24-h period when an individual's activity level reaches its peak. A delayed acrophase is often associated with eveningness (a preference for later sleep and activity times), while an advanced acrophase may correspond to morningness (a preference for earlier sleep and activity times).

To date, most RAR research has focused on adults, leaving significant gaps in understanding developmental variations and predictors of health outcomes in youth (Crowley et al., 2007). Prior research in adults indicates that RAR varies based on demographic factors (Li et al., 2021). For example, older adults typically exhibit more stable (higher IS) and less fragmented (lower IV) rest-activity patterns compared to younger adults, and males are likely to exhibit weaker RAR (lower IS and RA) than females (Wallace et al., 2023). Differences have also been found between and within various racial and ethnic identities (Hispanic, Non-Hispanic, White, Black, and Asian) (Cavaillès et al., 2025; Li et al., 2021; Potts et al., 2025). Because these dynamic patterns might change over time, identifying RAR metrics associated with adverse outcomes may guide clinicians as they tailor interventions for vulnerable populations (Chong et al., 2024).

RAR variables have also been shown to be associated with key indicators of physical health in adults. Weakened RAR patterns have been associated with increased cardiometabolic risk (higher IV and L5 and lower RA and IS) (Hoopes et al., 2021; Paewponsong et al., 2023), poorer sleep quality (Gonçalves et al., 2014), and diminished cognitive function (Kim et al., 2023; Smagula et al., 2019). Higher body mass index (BMI) and substance use are also associated with more fragmented and less stable RAR, as evidenced by lower IS, higher IV, and lower RA (Wallace et al., 2023). Together, these findings underscore RAR's utility as an objective biomarker for identifying individuals at risk for negative health outcomes in both clinical and population health contexts.

RAR has also been linked to psychological factors and mental health (Hooghiemstra et al., 2015; Meyrel et al., 2022). Irregular RAR, (i.e., lower IS and RA, higher IV, and a later acrophase) is linked to lifetime depressive and manic episodes (Smagula et al., 2018), and risk for psychosis. Specific RAR patterns in schizophrenia have been described, such as shorter active periods, lower RA, and lower M10 (Castro et al., 2015). Among older adults, less stable and more fragmented RAR is associated with dementia and cognitive decline (Smagula et al., 2019). Though more longitudinal work is necessary to determine changes over time, these findings suggest RAR metrics may have clinical value in detecting mental health risk.

Investigating RAR in adolescence is important due to the rapid physiological and psychological changes occurring in this developmental stage (Carskadon, 2011; Hagenauer and Lee, 2013). For example, adolescents commonly experience sleep disturbances driven by biological changes, such as delayed phase preference, coupled with increased social demands (Pifer et al., 2024). These disturbances negatively affect mood regulation, academic performance, and overall well-being (Crowley et al., 2007; Dewald et al., 2010; Owens et al., 2010). Examining RAR in adolescents may elucidate the complex relationships among sleep, physical activity, and health and allow for the identification of early biomarkers that may guide preventive interventions. This complex relationship in adolescence is also characterized by a developmental period in which circadian phase delay and psychosocial transitions are most prominent, therefore the age range was selected to align with the World Health Organization's definition of adolescence. The current scoping review therefore aims to document associations between RAR variables and demographic, social, physical, and psychological health characteristics from adolescence into young adulthood.

## 2 Methods

In this scoping literature review, we examined RAR variables most frequently reported in the literature, as well as demographic, social, physical, and psychological factors. Understanding these associations enables interpreting normative data and which metrics are best associated with negative outcomes and various diseases. See Table 1 for RAR variable descriptions. We also reviewed demographic characteristics, recording durations, devices used, and whether RAR analyses were parametric or non-parametric.

### 2.1 Design

This scoping review was conducted following the Preferred Reporting Items for Systematic Reviews and Meta-Analyses (PRISMA) guidelines. Articles were systematically screened, and data were extracted in accordance with PRISMA recommendations. Due to substantial heterogeneity in study designs, factors, and statistical analysis, findings were synthesized narratively as a scoping review rather than a systematic review or meta-analysis. Please see PROSPERO (CRD42023414974) for our published protocol.

### 2.2 Inclusion and exclusion criteria

We included studies published through April 2024 that reported RAR data in adolescent or young adult samples (ages 10–19). No start date restriction was applied to the search; all available articles at the time of search were considered. Cross-sectional or longitudinal studies published in peer-reviewed journals were eligible for inclusion if they: (1) provided data ranging from young adolescence to young adulthood (10–19 years old); (2) reported any statistical associations between RAR variables and demographic social, physical or psychological factors; and/or (3) included words such as “circadian rhythm” or “circadian activity”.

We excluded studies involving (1) pregnancy or in-utero assessments and (2) participants referenced as “adults” with no further age-related information. Studies were also excluded if the minimum value of the reported participant age range was above 18, if the mean participant age minus one standard deviation was greater than 18 (indicating that the lower bound of the age distribution was likely above 18), or if the mean participant age was above 25. The criterion based on mean age > 25 was applied only when age ranges or standard deviations were unavailable. In addition, we excluded protocol papers, abstracts, case reports, commentaries, letters, animal studies, and review articles.

### 2.3 Search strategy

**Figure 1:**
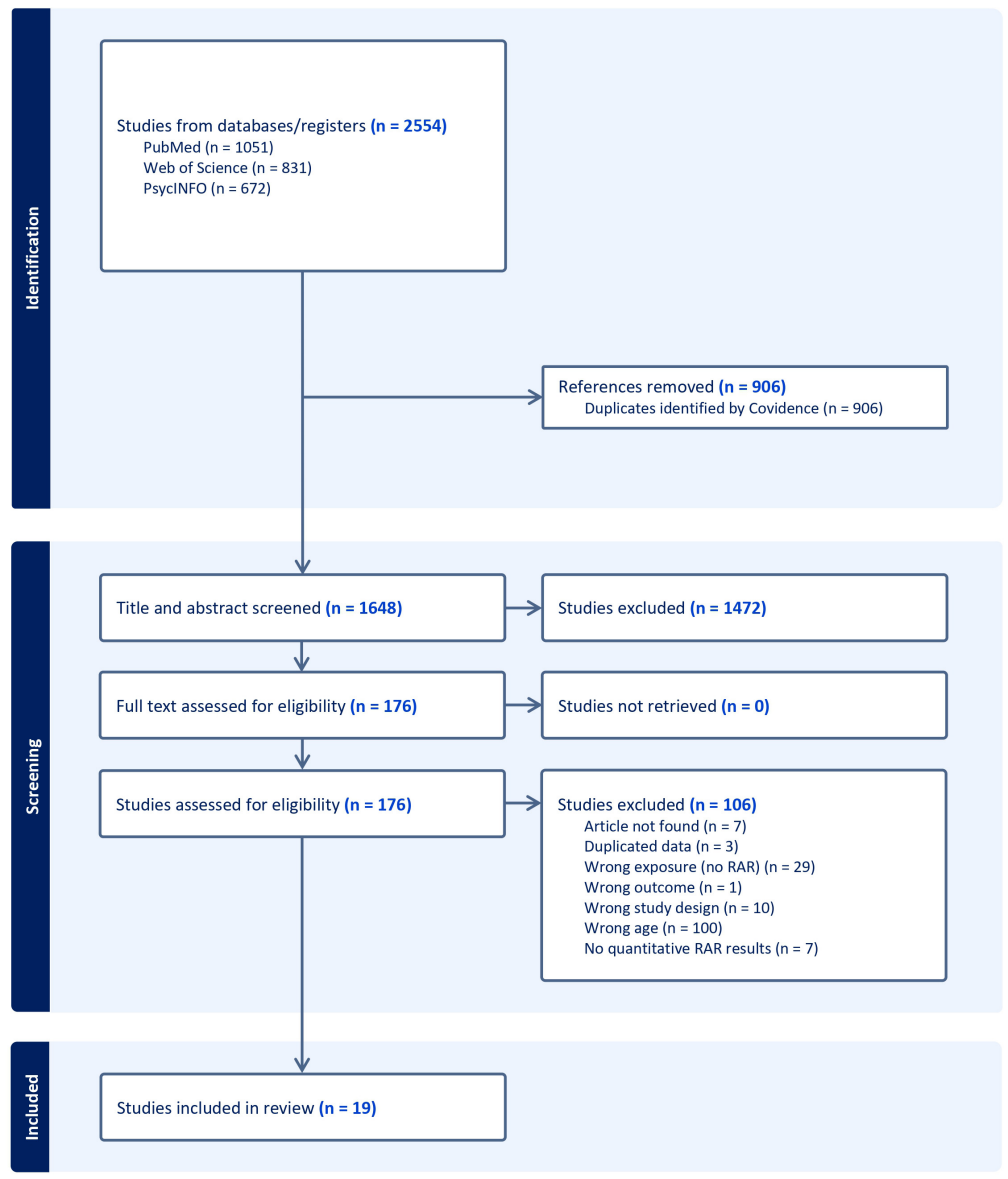
PRISMA flow chart of included studies.

The database search was conducted in April 2024 across PubMed, Web of Science, and PsycINFO, using keywords related to RAR, adolescents and young adults, and biopsychosocial outcomes. The complete search strategy is detailed in Supplementary Text 1. The initial search yielded 2,554 total studies: 1,051 from PubMed, 831 from Web of Science, and 672 from PsycINFO. After removing 906 duplicates, 1,648 studies underwent title and abstract screening, with 169 selected for full-text review. We excluded 157 studies in a full-text review, leaving a final sample of 19 studies included in this review. Please see Figure 1 for a detailed PRISMA flowchart and specific reasons for exclusion.

### 2.4 Paper screening and data extraction

All articles identified from our searches were imported into EndNote and subsequently uploaded to Covidence, a systematic review software designed to facilitate the review process and remove duplicates (Covidence Systematic Review Software, Veritas Health Innovation, 2024). Initially, titles and abstracts were screened for eligibility independently by two of four authors (e.g., CK, BN, DW, JK). Full texts of potentially eligible articles were similarly screened by two authors (e.g. CK, BN, DW, JK). Reasons for exclusion were documented during this stage. Disagreements regarding eligibility were resolved through discussion and consensus.

### 2.5 Data extraction and synthesis

Data extraction for all included studies (*k* = 19) was performed independently by two authors (CK and ES) under the supervision of senior authors (DW and JK). Synthesis of extracted data was performed and any discrepancies during data extraction were resolved through group consensus.

## 3 Results

### 3.1 Study and participants characteristics

Of the19 included studies, 14 were cross-sectional and 5 were prospective cohort studies collecting serial measurements of RAR. Nearly half of the studies (9/19) were conducted in the United States, followed by Brazil (3/19), Finland (2/19), Australia (2/19), and one study was conducted in multiple European countries (1/19). Detailed information on study locations is available in Supplementary Figure 1. Sample sizes ranged from 17 participants (Merikanto et al., 2017) to 12,526 participants (Wallace et al., 2023), with participant ages ranging from 10 to 25 years. The proportion of female participants varied from 0% (Merikanto et al., 2017) to 76% (Jääkallio et al., 2024). Only seven studies reported participant race and ethnicity. Studies were summarized according to the type of results reported and then classified by social/demographic, physical, or psychological factors. With respect to factors associated with RAR, seven studies reported social demographics factors (Blok et al., 2022; Grierson et al., 2016; Helena Study Group et al., 2017; Jääkallio et al., 2024; Mitchell et al., 2017; Rynders et al., 2022; Wallace et al., 2023), seven reported physical health factors (Chen et al., 2024; Helena Study Group et al., 2017; Quante et al., 2019a; Rechenberg et al., 2020; Rogers et al., 2020, 2021; Wallace et al., 2023), and ten reported psychological factors (Armitage et al., 2004; Blok et al., 2022; Castro et al., 2015; Grierson et al., 2016; Jääkallio et al., 2024; da Costa Lopes et al., 2023; Merikanto et al., 2017; Robillard et al., 2015; Tonon et al., 2022) (see Table 2 for a summary of the included studies).

**Table 2:** Description of the included studies.

Author, year	Type of analysis	N case	N controls	Total sample age: [range], mean, (SD)	Race: [group], population, *n* (%)	Ethnicity: [group], population, *n* (%)	Total % female	RAR variables	Days of wearable data collected
Armitage et al. (2004)	Difference	59	41	[12–17], 12.3 (2.9)	NA	NA	49	RA	5 days
Blok et al. (2022)	Association	788	344	[10–11], 11.67 (0.2), [13–14], 14.7 (0.33)		[10–11 years], Western, 716 (90.9%), non-Western, 72 (9.1%), [13–14 years], Western, 318 (92.4%), non-Western 26 (7.6%)	53	IS, IV, L5	9 days
Castro et al. (2015)	Difference, association	20	20	[13–27]	NA	NA	35	IS, IV, L5, M10, RA	15 days
Chen et al. (2024)	Difference, association	NA	18	[11–17], 14.6 (2.3)	NA	NA	56	RA, acrophase, Mesor	Range of 7–14 days
Grierson et al. (2016)	Difference, association	63	NA	[13–25]	NA	NA	62	RA, acrophase	Range of 5–14 days
Helena Study Group et al. (2017)	Difference, association	NA	1044	[12.5–17.5], 14.5, (1.2)	NA	NA	51	IV, M10, acrophase	7 days collection, 3 days of which have good quality or compliant data
Jääkallio et al. (2024)	Difference, association	233	NA	[16.8–18.4], 16.9, (0.6)	NA	NA	76	RA, acrophase, Mesor	Range of 7–10 days and nights
Lin et al. (2021)	Difference	20	14	NA	NA	NA	15	IS, IV, L5, M10, RA, acrophase, Mesor	14 days
da Costa Lopes et al. (2023)	Difference	85	NA	[15–17], 24.08, (5.61)	NA	NA	69	L5, M10, RA, acrophase, Mesor	7 days
Merikanto et al. (2017)	Descriptive	8	9	[14.5–17.5]	NA	NA	0	IS, IV, L5, M10, RA	Range of 25–44 days
Mitchell et al. (2017)	Difference, association	NA	58	11.6, (3.8)	White, 30 (51.7), Black or African American, 5 (8.6), Asian, 9 (15.5), Other, 14 (24.1)	NA	52	IS, IV, L5, M10, RA, acrophase, Mesor	7 days
Quante et al. (2019a)	Difference	209	567	[12.3–14.6], 13.2, (0.9)	White, 497 (64.0), Black 123 (15.8), Asian, 21 (2.7), Other, 101 (13.0)	Hispanic, 35 (4.5)	52	L5, M10, RA	Range of 7–10 days
Rechenberg et al. (2020)	Association	40	NA	13.4, (1.9)	NA	Non-White Hispanic, 30.8 (77.1)	60	RA, acrophase, Mesor	7 days
Robillard et al. (2015)	Difference	301	41	[12–35]	NA	NA	20	RA, acrophase	Range of 4–22 days
Rogers et al. (2020)	Difference, association	29	30	[13–18]	[Controls], White, 22 (73.3), Black/African American, 3 (10), non-black Hispanic, 4 (13.3), multiracial, 1 (3.3); [Survivors], White, 15 (51.7), Black/African American, 7 (24.1), non-black Hispanic, 4 (13.8), Asian, 2 (6.9), multiracial, 1 (3.4)	NA	61	RA, acrophase, Mesor	7 days
Rogers et al. (2021)	Difference, association	8	30	[13–18]	NA	NA	68	RA, acrophase, Mesor	21 days
Rynders et al. (2022)	Difference, association	16	NA	[14–19], 15.9, (1.2)	White, 14 (87.5)	Hispanic/Latino, 4 (25)	69	IS, IV, L5, M10	14 days
Tonon et al. (2022)	Difference, association	96	NA	[14–16]	NA	NA	56	IS, IV, L5, M10, RA, Mesor	14 days
Wallace et al. (2023)	Difference, association	NA	12526	[3–80], 40.93, (21.4)	Non-Hispanic Asian, 284 (5.81), non-Hispanic Black, 557 (13.13), non-Hispanic White, 745 (62.65), other Hispanic, 216 (6.25), other or multiracial 96 (3.35)	NA	51	IS, IV, L5, M10, RA	9 days

IS, interdaily stability; IV, intradaily variability; L5, lower 5 h of activity; M10, maximum 10 h of activity; MESOR, midline estimating statistic of rhythm; NA, not applicable; NS, not significant; RA, relative (circadian) amplitude; RAR, rest-activity rhythms; X: No; 3: Yes.

### 3.2 RAR variables characteristics

Studies reported the following RAR metrics, in order of descending frequency: acrophase (11/19), L5 (10/19), M10 (10/19), CA (10/19), IV (9/19), MESOR (9/19), IS (8/19), and RA (7/19). Six studies used parametric analysis, eight used non-parametric, and five used both methods.

### 3.3 Device characteristics

Device use varied across studies; the most used devices were Actiwatch (Philips^®^) (9/19) and ActiGraph^®^ (4/19). Other devices used included GENEActiv (ActivInsights^®^) (2/19), ActTrust (Condor^®^) (1/19), and MotionWatch (CamNtech^®^) (1/19). Participants were typically instructed to wear the actigraphy device on their non-dominant wrist (10/19) or according to the participant's preference/not specified (10/19). The epoch from the devices varied with most devices capturing it every minute (10/19). Other epoch lengths were 30 seconds (2/19) and 15 seconds (1/19). Eight studies did not specify the length of the epochs. Nine studies reported criteria for RAR data compliance (e.g., minimum wear time requirements, range from at least 10 to 16 h of wear time and/or minimum number of valid recording days needed for inclusion, ranging from 3 to 23 days), nine provided no such information, and one provided partial information.

### 3.4 Demographics and social factors

Among the 19 studies, 7 (36.84%) examined associations with demographic or social factors. The most frequently reported RAR metrics that were reported to have significant associations with these factors were IS (3/7), IV (3/7), M10 (2/7), MESOR (2/7), RA (2/7), and acrophase (2/7). Two studies compared age groups: both studies reported that younger adolescents exhibit higher IS and IV compared to older adolescents (Blok et al., 2022; Jääkallio et al., 2024; Mitchell et al., 2017). Three studies examined differences between males and females. All reported at least one significant difference by sex (Helena Study Group et al., 2017; Jääkallio et al., 2024; Wallace et al., 2023); however, the direction of association was not uniform. In the HELENA study males showed higher M10 but lower IV than females (Helena Study Group et al., 2017). In (Wallace et al., 2023) females showed higher IS and RA than males (Wallace et al., 2023). In contrast, Jääkallio et al. (2024) presented no sex differences were found in IS or IV but males had a higher MESOR (Jääkallio et al., 2024). The prior mentioned studies differed considerably in their design, Jääkallio et al. (2024) was a prospective cohort study, HELENA study is a cross-sectional, while Wallace et al., is an ecological cross-sectional study (Helena Study Group et al., 2017; Jääkallio et al., 2024; Wallace et al., 2023). See Table 3 for demographics and social results.

**Table 3:** Demographics and social factors: description of sub domains, case and control population, and RAR related findings.

Author, year	General population	Specific population	Case	Comparison/ controls	RAR association findings	RAR differences findings
Blok et al. (2022)	✓	X	10–11 yo (“younger”)	13–14 yo (“older”)	–	**IS**: older < younger **IV**: older < younger L5: ns
Grierson et al. (2016)	X	✓	Female	Male	*Age* CA: NS Acrophase: NS	**Gender** CA: NS Acrophase: NS
Helena Study Group et al. (2017)	✓	X	Female (F)	Male (M)	–	*Age***IV**: M < F **M10**: M > F **Acrophase**: M > F
Jääkallio et al. (2024)	✓	X	Female (F)	Male (M)	–	*Female vs. male time point 1 (T1)* CA: NS MESOR: NS *Female vs. male time point 2 (T2)* CA: NS MESOR: NS *RAR differences T1 vs. T2* CA: NS **MESOR**: T1 > T2
Wallace et al. (2023)	✓	X	13–19 yo	Male (M) vs. female (F)	–	**IS**: F > M IV: ns **L5**: M > F Start time L5: ns M10: ns Start time M10: ns **RA**: F > M
Rynders et al. (2022)	✓	X	Average age = 15.9 ± 1.2 years	Before vs. during COVID-19	–	*Weekday:* IS: ns IV: ns **Midpoint time L5**: before < during Mean counts L5: Midpoint time M10: ns Mean counts M10: ns RA: ns *Weekend:* IS: ns IV: ns Midpoint time L5: ns Mean counts L5: ns Midpoint time M10: ns Mean counts M10: ns RA: ns
Mitchell et al. (2017)	✓	X	10–14yo (“younger”)	15–18yo (“older”)	–	**IS**: younger > older **IV**: younger > older **Midpoint time L5**: Younger < older Mean counts L5: ns **Midpoint time M10**: younger < older Mean counts M10: ns

IS, interdaily stability; IV, intradaily variability; L5, lower 5 h of activity; M10, maximum 10 h of activity; MESOR, midline estimating statistic of rhythm; NR, not reported; NS, not significant; RA, relative (circadian) amplitude; RAR, rest-activity rhythms; yo: years old; X: no; ✓: yes.

### 3.5 Physical health factors

Nine studies reported RAR metrics relevant to physical health factors. The frequency of reported RAR variables across these studies were as follows: CA (7/9), MESOR (6/9), acrophase (6/9), M10 (4/9), IV (2/9), RA (2/9), IV (1/9), and IS (1/9). Among these, delayed acrophase (Chen et al., 2024; Helena Study Group et al., 2017), lower RA (Quante et al., 2019a), higher L5 (Quante et al., 2019a), lower M10 (Helena Study Group et al., 2017) and higher IV (Helena Study Group et al., 2017) were linked to poorer cardiometabolic outcomes, such as greater BMI, adiposity and reduced fitness. These findings diverged from those of Quante et al. which did not report a significant association between M10 and cardiometabolic health (Quante et al., 2019a), and from those of Rechenberg et al., which indicated no significant association between acrophase and cardiometabolic outcomes (Rechenberg et al., 2020). Most of these studies of cardiometabolic factors were cross-sectional, except for Lin et al. (2021) and Quante et al. (2019a),b, which used prospective designs (Lin et al., 2021; Quante et al., 2019a). Higher acrophase and M10 (da Costa Lopes et al., 2023) were associated with evening chronotype, and lower acrophase was associated with increased fatigue (Rogers et al., 2020). In contrast, Rechenberg et al. (2020) found no significant association between MESOR or acrophase and daytime sleepiness (Rechenberg et al., 2020). In participants with Kleine–Levin Syndrome, lower M10, MESOR, and RA were associated with hypersomnia attacks compared to both healthy controls and Kleine–Levin Syndrome participants without hypersomnia attack (Lin et al., 2021). See Table 4 for physical health reported results, including associative and difference findings.

**Table 4:** Physical health factors: description of sub domains, case and control population, and RAR related findings.

Author, year	General population	Specific population	Case	Comparison/ controls	RAR association findings	RAR differences findings
Chen et al. (2024)	✓	×	Adolescents	–	*Peak VO_2_* CA: ns MESOR: ns **Acrophase**: ↑acrophase → ↓ peak VO_2_	–
Grierson et al. (2016)	×	✓	Bipolar disorder	Unipolar disorder	*Body mass index (BMI)* CA: ns Acrophase: ns	–
Helena Study Group et al. (2017)	✓	×	Adolescents	–	*Weight* IV: ns **M10**: ↓M10 → ↑weight Acrophase: ns *Body mass index (BMI)* **IV**: ↑IV → ↑BMI **M10**: ↓M10 → ↑BMI Acrophase: ns *Waist* **IV**: ↑IV → ↑waist **M10**: ↓M10 → ↓waist **Acrophase**: ↑acrophase → ↑waist *Waist to height ratio (WHR)* **IV**: ↑IV → ↑WHR **M10**: ↓M10 → ↑WHR Acrophase: ns *Fat mass index (FMI): skinfolds, bioelectrical impedance analysis, DXA, dual-energy X-ray absorptiometry* **IV**: ↑IV → ↑FMI **M10**: ↓M10 → ↑FMI Acrophase: ns *Trunk fat (TF)* **IV**: ↑IV → ↑TF **M10**: ↓M10 → ↑TF	–
					Acrophase: ns *Cardiorespiratory fitness (CRF)* **IV**: ↑IV → ↓CRF **M10**: ↑M10 → ↑CRF Acrophase: ns *HDLc* **IV**: ↑IV → ↓HDLc **M10**: ↑M10 → ↑HDLc Acrophase: ns *Metabolic risk score 1: Anderson* **IV**: ↑IV → ↑Anderson score **M10**: ↑M10 → ↓Anderson score Acrophase: ns *Metabolic risk score 2: Martinez-Vizcaino (MV)* **IV**: ns **M10**: ↑M10 → ↓MV score Acrophase: ns	
Lin et al. (2021)	×	✓	Kleine–Levin syndrome (KLS) hyperinsomnia attack (attack-KLS), KLS no hyperinsomnia attack (no-KLS attack)	Controls	–	*Controls vs. attack-KLS and no-attack-KLS* **M10**: no-KLS attack > controls > attack-KLS L5: NS **RA**: no-KLS attack > controls > attack-KLS **CA**: no-KLS attack > attack-KLS = controls **MESOR**: no-KLS attack > controls > attack-KLS Acrophase: NS *Pre-attack phase (PAP) and early-attack phase (EAP) and late-attack phase (LAP) and recovery phase (RP)* IV: NS **IS:** RP > PAP ≈≈ EAP ≈ LAP **M10:** P ≈ PAP > LAP > EAP **L5:** LAP > EAP > RP > PAP **RA:** PAP > RP > LAP > EAP **MESOR:** RP > PAP > LAP > EAP **CA:** EAP > LAP > RP > PAP
da Costa Lopes et al. (2023)	×	✓	ValMet polymorphism	Val/Val	*Chronotype* **L5:** ↑L5 → ↑evening chronotype **M10**: ↑M10 → ↑evening chronotype CA: ns MESOR: ns **Acrophase**: ↑acrophase → ↑evening chronotype	*ValVal vs. ValMet* L5: ns M10: ns CA: ns MESOR: ns Acrophase: ns
Rogers et al. (2021)	×	✓	Cancer survivors	Controls	–	CA: ns MESOR: ns Acrophase: ns
Rogers et al. (2020)	×	✓	Cancer survivors	Controls	*Adolescent fatigue (survivors)* CA: ns MESOR: ns **Acrophase**: ↓acrophase, ↑fatigue *Adolescent fatigue (controls)* CA: ns MESOR: ns Acrophase: ns	*Survivors vs. controls:* CA: ns MESOR: ns Acrophase: ns
Rechenberg et al. (2020)	X	✓	Type 1 diabetes	NA	*Daytime sleepiness, executive function, stress/coping, self-care, glycemic control (glucose mean, variability, low/high indices), and diabetes QOL* CA: ns MESOR: ns Acrophase: ns	–
Quante et al. (2019a)	✓	×	NA	NA	*BMI:* **L5 count**: ↑BMI↑L5 L5 midpoint: ns M10 count: ns M10 midpoint: ns **RA**: ↑BMI↓RA *Waist circumference (WC):*	–
					**L5 count**: ↑WC↑L5 L5 midpoint: ns M10 count: ns M10 midpoint: ns **RA**: ↑WC↓RA *Subscapular ± triceps skinfold (STS):* **L5 count**: ↑STS↑L5 **L5 midpoint**: ↑STS↓L5 M10 count: ns M10 midpoint: ns **RA**: ↑STS↓RA *DXA total fat mass index (DXAtotal):* **L5 count**: ↑DXAtotal↑L5 L5 midpoint: ns M10 count: ns M10 midpoint: ns **RA**: ↑DXAtotal↓RA *DXA trunk fat mass index (DXAtrunk):* **L5 count**: ↑DXAtrunk↑L5 L5 midpoint: ns M10 count: ns M10 midpoint: ns **RA**: ↑DXAtrunk↓RA *Cardiometabolic risk (CR):* L5 count: ns L5 midpoint: ns M10 count: ns M10 midpoint: ns RA: ns	

BMI, body mass index; FMI, fat mass index; HDLc, high density lipoprotein cholesterol; IS, interdaily stability; IV, intradaily variability; L5, lower 5 h of activity; M10, maximum 10 h of activity; MESOR, midline estimating statistic of rhythm; NR, not reported; NA, not applicable; NS, not significant; RA, relative (circadian) amplitude; RAR, rest-activity rhythms; X: no; 3: yes.

### 3.6 Psychological factors

Ten studies reported RAR results relevant to psychological factors, with six investigating group differences between clinical and non-clinical groups across CA (4/6), IS (3/6), IV (3/6), L5 (3/6), M10 (3/6), acrophase (2/6), RA (2/6), and MESOR (1/6). Six studies of mood disorders found significantly lower CA (Armitage et al., 2004), higher acrophase (Robillard et al., 2015), and lower M10 onset (Merikanto et al., 2017) in those with depression compared to controls. Other studies indicated greater L5 and lower M10, RA, and MESOR in depressed adolescents compared to depression-risk groups (Tonon et al., 2022). Later acrophase was observed in bipolar disorder compared to in controls (Robillard et al., 2015), though those with depression did not differ from those with bipolar disorder in CA or acrophase (Grierson et al., 2016). In other disorders and risk groups, youth with anxiety had later acrophase than controls (Robillard et al., 2015), and youth with at-risk mental states had higher IS, lower IV and lower M10 compared to controls (Castro et al., 2015). Table 5 presents all reported results, including associative findings, related to psychological factors.

**Table 5:** Psychological health factors: description of sub domains, case and control population, and RAR related findings.

Author, year	General population	Specific population	Case	Comparison/ controls	RAR association findings	RAR differences findings
Armitage et al. (2004)	X	✓	Major depressive disorder (MDD)	Controls	–	**CA**: MDD < controls
Blok et al. (2022)	✓	X	10–11 years (“younger”)	13–14 years (“older”)	*Internalizing and externalizing problems: younger and older. dysregulation profile (DP): older* IS: ns IV: ns L5: ns *Dysregulation profile (DP): younger* IS: ns **IV**: ↑IV → ↑DP L5: ns	–
Castro et al. (2015)	X	✓	At-risk mental state (ARMS)	Controls	–	**IS:** ARMS > controls **IV:** ARMS < controls **M10:** ARMS < controls
Grierson et al. (2016)	X	✓	Bipolar disorder (BD)	Unipolar disorder (UD)	*Duration of illness (DI)* **CA**: ↑CA → ↓DI Acrophase: ns *Manic symptoms (MS)* CA: ns **Acrophase**: ↑CA → ↑MS *Depressive symptoms* CA: ns Acrophase: ns	*RAR variables did not differ between BD and UD grou*ps CA: ns Acrophase: ns
Jääkallio et al. (2024)	✓	X	Timepoint 1	Timepoint 2	*T1 affective problems: total sample, female, male* T2 CA: ns T2 MESOR: ns ↑T1 MESOR, ↑T2 MESOR in total sample, males, and females ↑T1 CA, ↑T2 CA in total sample, males, and females	–
da Costa Lopes et al. (2023)	X	✓	ValMet polymorphism	ValVal	*Attention* L5: ns M10: ns CA: ns MESOR: ns Acrophase: ns	–
Tonon et al. (2022)	X	✓	Major depressive disorder (MDD)	Low risk (LR) vs. high risk (HR)	Logistic regression predicting group membership (OR) *M10* HR vs. LR: ns MDD vs. LR: ns MDD vs. HR: ns *RA* **↑RA**↓ odds of HR vs. LR and MDD vs. LR	IS: ns IV: ns **L5**: (LR = HR) < MDD **M10:** LR < HR and MDD < HR **RA**: LR > HR > MDD **MESOR**: LR < HR > MDD Acrophase: ns
Rechenberg et al. (2020)	X	✓	Type 1 diabetes	NA	*Depressive symptoms; state anxiety:* CA: ns MESOR: ns Acrophase: ns *Trait anxiety (TA):* **CA**: ↑TA ↓CA **MESOR**: ↑TA ↓MESOR **Acrophase**: ↑TA ↓acrophase	–
Robillard et al. (2015)	X	✓	Psychiatric	Control vs. anxiety vs. depression vs. bipolar vs. psychosis	–	CA: NS **Acrophase**: anxiety, depression, bipolar > control
Merikanto et al. (2017)	X	✓	Depressed males	Controls	*Estimation of group membership with RAR variable:* IS: ns IV: ns L5: ns L5 onset: ns M10: ns **M10 onset**: ↑depression ↓M10 onset RA: ns **CA**: ↑depression ↓CA	*Depressed vs. controls* IS: ns IV: ns L5: ns L5 onset: ns M10: ns **M10 onset**: depressed < controls RA: ns CA: ns

IS, interdaily stability; IV, intradaily variability; L5, lower 5 h of activity; M10, maximum 10 h of activity; MESOR, midline estimating statistic of rhythm; NR, not reported; NS, not significant; RA, relative (circadian) amplitude; RAR, rest-activity rhythms; X: no; 3: yes.

## 4 Discussion

This scoping review synthesized 19 studies of RAR in adolescents and young adults to describe reported associations between RAR and demographic, physical health, and psychological factors. Among these studies, nine included participants from the general population and ten included clinical disease samples. Around 37% examined findings of demographic or social factors, 47% physical health, and 53% psychological factors. The sample size of the studies varied considerably, ranging from 17 to over 12,000 individuals, with the largest study drawing from a National-level data repository. Consistent findings showed that younger age groups exhibited higher IS and IV, and that elevated cardiometabolic risk was associated with a delayed acrophase (increased acrophase). Overall associations between RAR metrics and demographic, physical, and psychological factors were inconsistent compared to adult literature and within the included studies. This inconsistency may be due to differences in study designs and populations, absence of standardized methods to calculate RAR metrics (e.g. parametric vs. non-parametric) and evaluate RAR data quality (e.g. minimum wear time requirements and/or minimum number of valid recording days needed for inclusion).

### 4.1 RAR, demographic variations and developmental trajectories

Studies found evidence for age-associated RAR differences across adolescents and young adults. Three studies investigating age differences in youth found lower stability (IS) and lower fragmentation (IV) in older adolescents compared to younger ones (Blok et al., 2022; Mitchell et al., 2017), which contrasts with adults who typically demonstrate inversely associated IS and IV; other studies also found older adolescents had higher MESOR and later L5 midpoint than younger ones (Jääkallio et al., 2024; Mitchell et al., 2017; Rynders et al., 2022). One of these studies also found several other significant differences between younger vs. older adolescents, including that older adolescents had later acrophase, later midpoint M10, and higher RA (Mitchell et al., 2017). Still, across the literature, associations in two studies did not reach statistical significance, including no IS or IV differences in youth before or during COVID (Rynders et al., 2022), and no age differences in CA or acrophase between youth with bipolar vs. unipolar depression (Grierson et al., 2016).

Generally, these developmental patterns contrast with adulthood trajectories, where aging is typically associated with earlier circadian timing and greater stability (Li et al., 2021). Thus, while adult RAR patterns trend toward consolidation and morningness with advancing age, adolescence appears to reveal increasing irregularity and eveningness. This developmental divergence is likely driven by pubertal changes, including circadian phase delay and changing social demands and overall decline in physical activity among adolescents characteristic of this developmental window (Allison et al., 2007; Pifer et al., 2024). While these age-specific differences in RAR during adolescence may reflect normal developmental processes, when extreme, they could signal risk for adverse outcomes, suggesting the importance of age-appropriate normative data and intervention approaches tailored to adolescent physiology.

Sex differences in RAR also emerged inconsistently across studies. For example, one study reported stronger RAR patterns (higher IS and RA and lower L5) in females compared to males (Wallace et al., 2023), whereas another study found higher IV, lower M10, and lower acrophase in females than in males (Helena Study Group et al., 2017). Some studies also found no such gender differences. That is, Jääkallio et al. (2024) reported no sex differences in CA or MESOR (Jääkallio et al., 2024). These observed inconsistent patterns differ from adult studies, where women generally demonstrate stronger and more regular rhythms (Wallace et al., 2023), suggesting that sex-linked RAR differences may not fully establish until later development.

A critical gap identified in this review is the limited examination of racial and ethnic differences in youth RAR patterns. Only seven studies reported race and/or ethnicity data, despite evidence from adult populations showing significant disparities and the emergence of differences during adolescence and early adulthood (Li et al., 2021; Wallace et al., 2023). Adult research demonstrates that Hispanic individuals generally exhibit stronger and more stable RAR patterns (higher CA, higher MESOR, lower IV), while non-Hispanic Black individuals show weaker and less stable patterns (Li et al., 2021), though variability within ethnic groups also exists (Cavaillès et al., 2025; Potts et al., 2025). The absence of diverse adolescent samples represents a significant limitation in understanding how health disparities in RAR may emerge during development and persist across the lifespan.

### 4.2 Physical health associations

Findings related to RAR metrics and physical health are mixed in adolescents and young adults. Delayed acrophase (Helena Study Group et al., 2017; Lin et al., 2021), lower RA (Quante et al., 2019a), higher L5 (Quante et al., 2019a), lower M10 (Helena Study Group et al., 2017) and higher IV (Helena Study Group et al., 2017) have each been associated with poorer cardiometabolic outcomes, including higher BMI, greater adiposity and reduced fitness. In contrast to the HELENA Study (Helena Study Group et al., 2017), Quante et al. (2019a),b found no association between M10 and cardiometabolic health (Quante et al., 2019a), and unlike Chen et al. (2024) and HELENA Study (Helena Study Group et al., 2017), Rechenberg et al. (2020) reported no association between acrophase and cardiometabolic outcomes (Rechenberg et al., 2020). Studies in the adult population report a clearer pattern: Li et al. (2022) associated higher IV with obesity and lower amplitude, MESOR, and IS, and Hoopes et al. (2021) and Makarem et al. (2024) identified lower IS and higher IV as biomarkers of cardiometabolic risk. Across both adults and adolescent's studies, higher IV appears to be a consistent indicator of elevated cardiometabolic risk, which reflects a more fragmented 24-h rhythm with frequent awakenings during the night and irregular daytime activity. Whereas IV was identified as a biomarker for both age groups, in adults, lower IS appeared as a risk indicator, reflecting that a less regular day-to-day activity pattern (e.g., irregular bed/wake times, variable daytime activity, shift work) may impact cardiometabolic outcomes. Strategies that increase IS (greater day-to-day regularity) and reduce IV (less within-day fragmentation) may therefore be metabolically beneficial. These findings support that irregular routines may implicate in poorer cardiometabolic outcomes, the social zeitgebers hypothesis supports the role of stable social routines (timed light, meals, activity, social contacts) in synchronizing these rhythms, offering plausible behavioral levers for improving RAR profiles (Ehlers et al., 1988; Quante et al., 2019b).

Evidence on sleep and fatigue is similarly inconsistent in adolescents. Among youth carrying the Val polymorphism, an evening chronotype has been associated with a later acrophase, higher M10 and higher L5 (da Costa Lopes et al., 2023). The Val66Met polymorphism in the BDNF gene is associated with reduced circulating BDNF (Egan et al., 2003) and has been linked to poorer cognitive performance (Avgan et al., 2017), as well as differences in chronotype and greater social jetlag (Saitoh et al., 2018). In pediatric oncology samples, greater fatigue has been tied to a lower acrophase (Rogers et al., 2020) whereas daytime sleepiness showed no association with MESOR or acrophase (Rechenberg et al., 2020). In Kleine–Levin syndrome, episodes of hypersomnia have been linked to lower M10, MESOR, and RA (Lin et al., 2021)—findings that run counter to results reported in other populations. These discrepancies likely reflect the inclusion of condition-specific studies with diverse RAR patterns, implying that the association of RAR, sleep and fatigue features may be clinically informative in disorder-specific ways. Characterizing RAR more precisely could enable targeted interventions to regularize rhythms personalized for each clinical condition; for example, bright-light strategies have improved adolescent circadian timing (Richardson et al., 2018), which by consequence may improve daytime sleepiness and fatigue throughout the day (Hong et al., 2021).

### 4.3 Psychological associations

In adolescent studies, poor mental health has been associated with lower M10 (reduced peak daytime activity) (Castro et al., 2015; Merikanto et al., 2017; Tonon et al., 2022), while other RAR metrics were typically examined in isolation, limiting any conclusion about their consistency across conditions. In mood disorder studies, findings include lower CA (Armitage et al., 2004), higher acrophase (Robillard et al., 2015), higher L5 (Tonon et al., 2022), lower RA (Tonon et al., 2022), and lower MESOR (Tonon et al., 2022). Among adults, lower IS and RA, higher IV, and a later acrophase have been associated with depressive and manic symptoms (Smagula et al., 2018), in contrast with our results. Beyond mood disorders, RAR metrics have again been reported in isolation: anxious youth showed higher acrophase than controls (Robillard et al., 2015), and youth with at-risk mental states displayed higher IS, lower IV, and lower M10 vs. controls (Castro et al., 2015). In adults, anxiety has been linked to higher IV but not IS (Luik et al., 2015), and to our knowledge acrophase has not been reported as associated with anxiety. No adult studies have evaluated at-risk mental states in relation to RAR. To conclude, lower M10 was frequently observed across mental health conditions (Iob et al., 2023), it indicates that people tend to move less during their “most active” hours (reduced peak daytime activity). Physical activity is linked to better mental health: meta-analyses show that higher daily step counts correlate with fewer depressive symptoms (Bizzozero-Peroni et al., 2024). Thus, encouraging movement throughout the day, especially during peak activity periods, may therefore support mental health.

### 4.4 Methodological considerations and limitations

Studies varied widely in devices used (Actiwatch, ActiGraph, GENEActiv), epoch lengths (15 s to 1 min), recording durations (5–44 days), and RAR data compliance (i.e., minimum wear time requirements and/or minimum number of valid recording days needed for inclusion). While 47.4% of studies reported data compliance criteria, 47.4% provided no such information. Across devices, accelerometer sensors can capture movement as triaxial or uniaxial, using different softwares to process RAR data. Because we did not collect these specifications of the devices used in the studies, our ability to interpret the inconsistent findings is limited. Differences in devices themselves, analytic approaches (parametric vs. non-parametric), and study quality may also further complicate synthesis. The heterogeneity of studies included in the current review precludes generalizable conclusions across these factors.

The predominance of cross-sectional designs (73.7% of studies) further limits causal inference. Most investigations examined associations at single time points, precluding understanding of how RAR patterns change during adolescent development or whether disruptions precede adverse outcomes. Longitudinal studies bridging adolescence into adulthood are critically needed to chart developmental trajectories and establish predictive validity of RAR metrics.

Sample sizes ranged considerably from 17 to 12,526 participants, with some studies potentially underpowered to detect significant associations. Additionally, the geographic distribution was heavily weighted toward high-income countries (47.4% from the United States), limiting generalizability to global adolescent populations. Further, both general and specific populations were included in this review, which can limit comparison with adults and constrain the conclusions that can be drawn about biopsychosocial factors.

### 4.5 Future research directions

Several key priorities emerge from this review. Mechanistic studies are needed to clarify the biological pathways linking RAR disruptions to health outcomes, including the role of hormonal regulation, inflammation, and neuroplasticity (Armitage et al., 2004). The increasing availability of consumer wearables offers opportunities for large-scale, longitudinal monitoring of RAR patterns, but validation studies in adolescent populations are required to establish clinical utility. Randomized controlled trials testing interventions to strengthen RAR patterns could establish causal relationships and inform clinical practice, particularly trials of structured activity programs, light therapy, and behavioral sleep interventions. Future research should also examine RAR alongside other biomarkers, such as hormonal or inflammatory markers and neuroimaging, to develop integrative models of adolescent health risk. Finally, environmental and social determinants—including light exposure, school schedules, social media use, and peer or family dynamics—should be studied to understand how external contexts shape RAR during adolescence.
