# Supplementary material for: Patterns of rest-activity rhythms from adolescence to young adulthood: a scoping review
Source: Front Sleep. 2026 Apr 8;5:1779808. doi: 10.3389/frsle.2026.1779808 (PMC13099328; doi:10.3389/frsle.2026.1779808)
Supplement: Supplementary file 1 [file Data_Sheet_1.docx]

Supplementary Material

# Supplementary Text 1. Search Strategy

| **Search Ter**  **ms** | **# of Hits** |
| --- | --- |
| ("rest-activity rhythms") AND ("child" OR "adolescent") | 23 |
| ("rest-activity rhythms" OR "L5" OR "M10" OR "circadian period" OR "relative amplitude" OR "amplitude" or "midline-estimating statistic of rhythm" OR "acrophase" OR "intraindividual variability" OR "interday stability" OR "intraday variability") AND ("child" OR "adolescent") | 19,675 |
| ("rest-activity rhythms" OR "L5" OR "M10" OR "circadian period" OR "midline-estimating statistic of rhythm" OR "acrophase") AND ("child" OR "adolescent") | 2,579 |
| ("actigraphy" OR "wearable") AND ("rest-activity rhythms" OR "L5" OR "M10" OR "circadian period" OR "midline-estimating statistic of rhythm" OR "acrophase") AND ("child" OR "adolescent") | 56 |
| ("actigraphy" OR "wearable") AND ("rest-activity rhythms" OR "L5" OR "M10" OR "circadian period" OR "midline-estimating statistic of rhythm" OR "acrophase") AND ("sleep" OR "circadian") AND ("child" OR "adolescent") | 53 |
| ("actigraphy" OR "wearable") AND ("rest-activity rhythms" OR "L5" OR "M10" OR "circadian period" OR "relative amplitude" OR "amplitude" or "midline-estimating statistic of rhythm" OR "acrophase" OR "intraindividual variability" OR "interday stability" OR "intraday variability") AND ("sleep" OR "circadian") AND ("child" OR "adolescent") | 92 |

| ("actigraph"[All Fields] OR "actigraphy"[All Fields] OR "wearable"[All Fields] OR "wrist-worn"[All Fields] OR "accelerometer"[All Fields] OR "accelerometry"[All Fields]) AND ("child"[All Fields] OR "children"[All Fields] OR "kids"[All Fields] OR "teens"[All Fields] OR "adolescence"[All Fields] OR "adolescents"[All Fields] OR "young adults"[All Fields] OR "pediatric"[All Fields]) AND ("rest-activity"[All Fields] OR "parametric"[All Fields] OR "nonparametric"[All Fields] OR "rhythm"[All Fields] OR "L5"[All Fields] OR "M10"[All Fields] OR "interdaily stability"[All Fields] OR "intradaily variability"[All Fields]) | 519 |
| --- | --- |

Age terms:

TI=(adolesc* OR teen* OR youth? OR juvenile* OR (young* NEAR (adult* OR person* OR people OR patient*))) OR AK=(adolesc* OR teen* OR youth? OR juvenile* OR (young* NEAR (adult* OR person* OR people OR patient*))) OR AK=(adolesc* OR teen* OR youth? OR juvenile* OR (young* NEAR (adult* OR person* OR people OR patient*)))

| ("actigraph"[All Fields] OR "actigraphy"[All Fields] OR "wearable"[All Fields] OR "wrist-worn"[All Fields] OR "accelerometer"[All Fields] OR "accelerometry"[All Fields]) AND (adolesc* OR teen* OR youth? OR juvenile* OR pediatric OR kid OR teen OR (young* NEAR (adult* OR person* OR people OR patient*))) AND ("rest-activity"[All Fields] OR "parametric"[All Fields] OR "nonparametric"[All Fields] OR "rhythm"[All Fields] OR "L5"[All Fields] OR "M10"[All Fields] OR "interdaily stability"[All Fields] OR "intradaily variability"[All Fields]) | 551 |
| --- | --- |

| ("actigraph"[All Fields] OR "actigraphy"[All Fields] OR "wearable"[All Fields] OR "wrist-worn"[All Fields] OR "accelerometer"[All Fields] OR "accelerometry"[All Fields]) AND (adolesc* OR teen* OR youth? OR juvenile* OR pediatric OR kid OR child OR children OR infant OR teen OR (young* OR emerging* NEAR (adult* OR person* OR people OR patient*))) AND ("rest-activity"[All Fields] OR "parametric"[All Fields] OR "nonparametric"[All Fields] OR "rhythm"[All Fields] OR "L5"[All Fields] OR "M10"[All Fields] OR "interdaily stability"[All Fields] OR "intradaily variability"[All Fields]) | 960 |
| --- | --- |

#now we get the Qian paper:

| (actigraph*[All Fields] OR "actigraphy"[All Fields] OR wearable*[All Fields] OR "wrist-worn"[All Fields] OR acceleromet*[All Fields]) AND (child*[All Fields] OR "kids"[All Fields] OR teen*[All Fields] OR "adolescence"[All Fields] OR "adolescents"[All Fields] OR "young adults"[All Fields] OR "pediatric"[All Fields]) AND ("rest-activity"[All Fields] OR (activ* rhythm*[All Fields]) OR "activity rhythms"[All Fields] OR "acrophase"[All Fields] OR "midline-estimating"[All Fields] OR "parametric"[All Fields] OR "nonparametric"[All Fields] OR rhythm*[All Fields] OR "L5"[All Fields] OR "M10"[All Fields] OR "interdaily stability"[All Fields] OR "intradaily variability"[All Fields]) | 638 |
| --- | --- |

With young adult/emerging adult terms (now we get the Hoopes paper):

| (actigraph*[All Fields] OR "actigraphy"[All Fields] OR wearable*[All Fields] OR "wrist-worn"[All Fields] OR acceleromet*[All Fields]) AND (child*[All Fields] OR "kids"[All Fields] OR teen*[All Fields] OR "adolescence"[All Fields] OR "adolescents"[All Fields] OR (young adult*[All Fields]) OR (emerg* adult*[All Fields]) OR "pediatric"[All Fields]) AND ("rest-activity"[All Fields] OR (activ* rhythm*[All Fields]) OR "activity rhythms"[All Fields] OR "acrophase"[All Fields] OR "midline-estimating"[All Fields] OR "parametric"[All Fields] OR "nonparametric"[All Fields] OR rhythm*[All Fields] OR "L5"[All Fields] OR "M10"[All Fields] OR "interdaily stability"[All Fields] OR "intradaily variability"[All Fields]) | 1,009 |
| --- | --- |

With young adult/emerging adult terms AND more infant-specific terms:

| (actigraph*[All Fields] OR "actigraphy"[All Fields] OR wearable*[All Fields] OR "wrist-worn"[All Fields] OR acceleromet*[All Fields]) AND (child*[All Fields] OR infan*[All Fields] OR newborn*[All Fields] OR “babies”[All Fields] OR “baby”[All Fields] OR "kids"[All Fields] OR teen*[All Fields] OR "adolescence"[All Fields] OR "adolescents"[All Fields] OR (young adult*[All Fields]) OR (emerg* adult*[All Fields]) OR "pediatric"[All Fields]) AND ("rest-activity"[All Fields] OR (activ* rhythm*[All Fields]) OR "activity rhythms"[All Fields] OR "acrophase"[All Fields] OR "midline-estimating"[All Fields] OR "parametric"[All Fields] OR "nonparametric"[All Fields] OR rhythm*[All Fields] OR "L5"[All Fields] OR "M10"[All Fields] OR "interdaily stability"[All Fields] OR "intradaily variability"[All Fields]) | 1,046 |
| --- | --- |

A search strategy should result in these papers being found:

- Armitage R, Hoffmann R, Emslie G, Rintelman J, Moore J, Lewis K. Rest-activity cycles in childhood and adolescent depression. J Am Acad Child Adolesc Psychiatry. 2004 Jun;43(6):761-9. doi: 10.1097/01.chi.0000122731.72597.4e. PMID: 15167093.
- Beunders VAA, Koopman-Verhoeff ME, Vermeulen MJ, Silva CCV, Jansen PW, Luik AI, Reiss IKM, Joosten KFM, Jaddoe VWV. Fetal and infant growth patterns, sleep, and 24-h activity rhythms: a population-based prospective cohort study in school-age children. J Sleep Res. 2023 Aug;32(4):e13822. doi: 10.1111/jsr.13822. Epub 2023 Jan 27. PMID: 36707974.
- Beunders VAA, Koopman-Verhoeff ME, Vermeulen MJ, Jansen PW, Luik AI, Derks IPM, Reiss IKM, Joosten KFM, Jaddoe VWV. Sleep, 24-hour activity rhythms, and cardiometabolic risk factors in school-age children. J Clin Sleep Med. 2023 Jul 1;19(7):1219-1229. doi: 10.5664/jcsm.10544. PMID: 36866620; PMCID: PMC10315610.
- Mitchell JA, Quante M, Godbole S, James P, Hipp JA, Marinac CR, Mariani S, Cespedes Feliciano EM, Glanz K, Laden F, Wang R, Weng J, Redline S, Kerr J. Variation in actigraphy-estimated rest-activity patterns by demographic factors. Chronobiol Int. 2017;34(8):1042-1056. doi: 10.1080/07420528.2017.1337032. Epub 2017 Jun 26. PMID: 28650674; PMCID: PMC6101244.
- Qian J, Martinez-Lozano N, Tvarijonaviciute A, Rios R, Scheer FAJL, Garaulet M. Blunted rest-activity rhythms link to higher body mass index and inflammatory markers in children. Sleep. 2021 May 14;44(5):zsaa256. doi: 10.1093/sleep/zsaa256. PMID: 33249510; PMCID: PMC8120335.
- Quante M, Cespedes Feliciano EM, Rifas-Shiman SL, Mariani S, Kaplan ER, Rueschman M, Oken E, Taveras EM, Redline S. Association of Daily Rest-Activity Patterns With Adiposity and Cardiometabolic Risk Measures in Teens. J Adolesc Health. 2019 Aug;65(2):224-231. doi: 10.1016/j.jadohealth.2019.02.008. Epub 2019 May 2. PMID: 31056236; PMCID: PMC6650322.
- Wallace DA, Johnson DA, Redline S, Sofer T, Kossowsky J. Rest-activity rhythms across the lifespan: cross-sectional findings from the US representative National Health and Nutrition Examination Survey. Sleep. 2023 Nov 8;46(11):zsad220. doi: 10.1093/sleep/zsad220. PMID: 37610882; PMCID: PMC10636247.

#
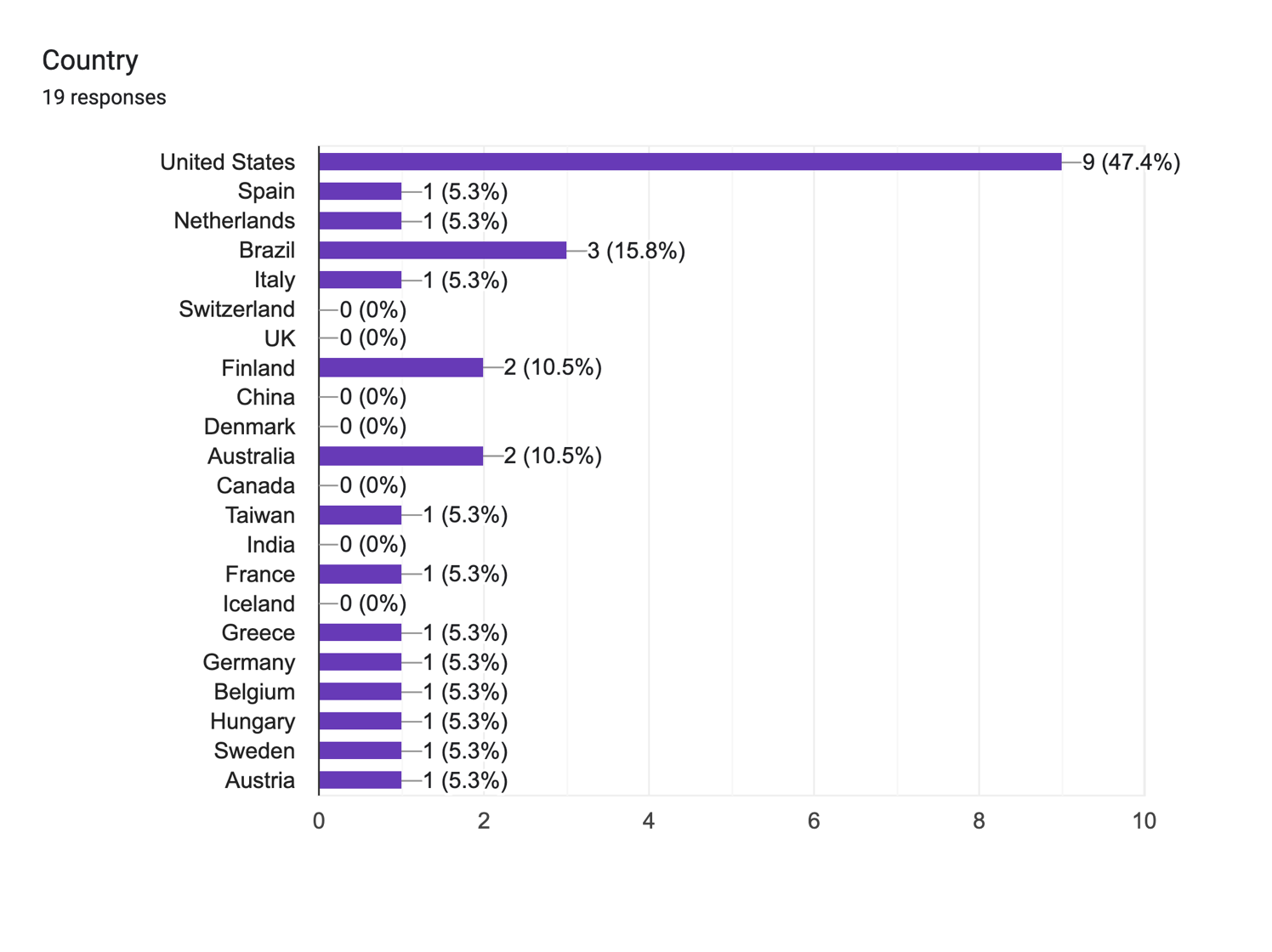


**Supplementary Figure 1. Location of included studies**
